# The combination of G-CSF and AMD3100 mobilizes bone marrow-derived stem cells to protect against cisplatin-induced acute kidney injury in mice

**DOI:** 10.1186/s13287-021-02268-y

**Published:** 2021-03-24

**Authors:** Zhi Chen, Xiang Ren, Ruimin Ren, Yonghong Wang, Jiwen Shang

**Affiliations:** 1 Department of Geriatrics, Tongji Hospital, Tongji Medical College, Huazhong University of Science and Technology, Wuhan, 430030 China; 2 Department of Urology, Third Hospital of Shanxi Medical University, Shanxi Bethune Hospital, Shanxi Academy of Medical Sciences, Taiyuan, 030032 China; 3 Tongji Shanxi Hospital, Tongji Medical College, Huazhong University of Science and Technology, Taiyuan, 030032 China; 4 Department of Neurosurgery, Third Hospital of Shanxi Medical University, Shanxi Bethune Hospital, Shanxi Academy of Medical Sciences, Taiyuan, 030032 China; 5 Department of Ambulatory Surgery, Third Hospital of Shanxi Medical University, Shanxi Bethune Hospital, Shanxi Academy of Medical Sciences, No. 99 Longcheng Street, Taiyuan, 030032 Shanxi China

**Keywords:** Cisplatin nephrotoxicity, AMD3100, Chemokine, Acute kidney injury

## Abstract

**Background:**

Several studies have confirmed that mobilizing bone marrow-derived stem cells (BMSCs) ameliorates renal function loss following cisplatin-induced acute kidney injury (AKI). The aim of this study was to explore whether the combination of granulocyte-colony stimulating factor (G-CSF) and plerixafor (AMD3100) exerts beneficial effects on renal function recovery in a model of cisplatin-induced nephrotoxicity.

**Methods:**

C57BL/6J mice received intraperitoneal injections of G-CSF (200 μg/kg/day) for 5 consecutive days. On the day of the last injection, the mice received a single subcutaneous dose of AMD3100 (5 mg/kg) 1 h before cisplatin 20 mg/kg injection. Ninety-six hours after cisplatin injection, the mice were euthanized, and blood and tissue samples were collected to assess renal function and tissue damage. Cell mobilization was assessed by flow cytometry (FCM).

**Results:**

Mice pretreated with G-CSF/AMD3100 exhibited longer survival and lower serum creatinine and blood urea nitrogen (BUN) levels than mice treated with only G-CSF or saline. Combinatorial G-CSF/AMD3100 treatment attenuated tissue injury and cell death, enhanced cell regeneration, and mobilized a higher number of stem cells in the peripheral blood than G-CSF or saline treatment. Furthermore, the mRNA expression of proinflammatory factors was lower, whereas that of anti-inflammatory factors was higher, in the G-CSF/AMD3100 group than in the G-CSF or saline group (all *P* < 0.05).

**Conclusions:**

These results suggest that combinatorial G-CSF/AMD3100 therapy mobilizes BMSCs to accelerate improvements in renal functions and prevent cisplatin-induced renal tubular injury. This combinatorial therapy may represent a new therapeutic option for the treatment of AKI and should be further investigated in the future.

## Background

The two most common causes of acute kidney injury (AKI) are ischaemia/reperfusion (I/R) injury and nephrotoxic agent exposure [1]. Cisplatin is a chemotherapeutic drug widely administered to treat various solid tumours. Nephrotoxicity is a severe side effect of cisplatin administration and often results in acute renal disease [2]. Cisplatin induces renal damage in 25 to 35% of patients, and the curative effects and nephrotoxicity of cisplatin are dose-dependent. Cisplatin-induced toxic nephropathy is typically characterized by tubular necrosis and apoptosis and greatly limits the use of this agent in clinical settings. Hence, novel therapies for cisplatin-induced AKI that do not compromise the anti-tumour effects of cisplatin are needed.

Stem cell therapy has attracted great interest as a treatment for AKI. Numerous studies have demonstrated that stem cells can repair damaged tubular cells and attenuate cisplatin-induced AKI [3, 4]. Bone marrow-derived stem cells (BMSCs) can transdifferentiate into endothelial and epithelial cells [5]. Several studies have demonstrated that mobilizing BMSCs to treat AKI in animal models facilitates significant improvements in renal function and enables the repair of renal tissue structural damage [6, 7]. Granulocyte colony-stimulating factor (G-CSF) mobilizes BMSCs to sites of renal injury, where these cells transdifferentiate into renal stem cells [6, 8]. The CXCR4 antagonist plerixafor (AMD3100) interferes with the interaction of CXCR4 with its natural ligand, SDF-1, and mobilizes CD34^+^ stem cells from the bone marrow into the peripheral blood [9]. It exerts beneficial effects against I/R-induced AKI [10] and myocardial infarction [11]. However, several studies have reported that continuous AMD3100 administration accelerates renal function decline and exerts adverse effects on renal tissue repair [12, 13]. Theiss et al. demonstrated that at high concentrations, AMD3100 mobilizes BMSCs, whereas at low concentrations, AMD3100 does not mobilize BMSCs and does not improve survival in the setting of myocardial infarction [14]. However, Zuk et al. demonstrated that a single dose of 5 mg/kg AMD3100 was less effective at mobilizing BMSCs than a single dose of 1 mg/kg AMD3100 in a rat model of renal I/R [15]. Other studies have demonstrated that the combination of G-CSF and AMD3100 better increases BMSC mobilization than G-CSF alone [16, 17]. Therefore, the usefulness and optimal dosage of AMD3100 remain controversial. In addition, few studies have assessed the combination of G-CSF and AMD3100 as a therapy for cisplatin-induced AKI.

Based on the above findings, we speculated that combinatorial therapy with G-CSF/AMD3100 may be more effective in recruiting BMSCs into renal tissue to repair and ameliorate cisplatin-induced AKI than either therapy alone. In addition, the mechanisms underlying the renoprotective effects of BMSCs require further elucidation. In the present study, we first demonstrated that G-CSF/AMD3100 mobilizes BMSCs into the peripheral blood and prevents cisplatin-induced AKI in mice. Second, we demonstrated that mobilization of BMSCs by G-CSF/AMD3100 decreases tubular cell apoptosis and promotes proliferation. The protective effects of this combination therapy are associated with a reduction in the levels of putative biomarkers of renal injury and inflammation.

## Materials and methods

### Ethics statement

All experiments were performed using C57BL/6J mice and were conducted according to the Huazhong University of Science and Technology Guide for the Care and Use of Laboratory Animals. All experimental animal procedures were approved by the Institutional Animal Care and Use Committee of Huazhong University of Science and Technology, Wuhan, China.

### Animals

Experiments were performed with 7- to 8-week-old male C57BL/6 mice (weighing 20–25 g) purchased from Hua Fukang Company (Beijing, China). The animals were housed according to the institutional animal research guidelines and were maintained under constant temperature, a 12-h light/dark cycle, and 50 ± 5% humidity with standard mouse chow and water available ad libitum.

### Cisplatin-induced AKI model and treatment protocol

**Fig. 1:**
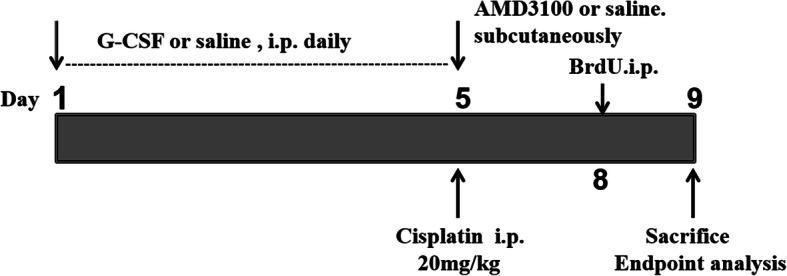
Timeline of the experimental design of this study. Seven- to 8-week-old male C57BL/6 mice were divided into four groups. All mice received G-CSF (200 μg/kg/d) for 5 consecutive days and received subcutaneously a signal AMD3100 (5 mg/kg) 1 h before an intraperitoneal injection of cisplatin 20 mg/kg and injections of 5-bromo-2-deoxyuridine (BrdU) (100 mg/kg/day) for 2 consecutive days from the indicated time

The experimental design is shown in Fig. 1. AKI was induced in C57BL/6J mice via intraperitoneal injection of 20 mg/kg body weight cisplatin (Sigma Chemical Co.) which was dissolved in saline. Mice were randomly allocated to the following groups: the untreated cisplatin (control) group (*n* = 12), cisplatin (Cis, 20 mg/kg) + saline group (*n* = 12), Cis + G-CSF (200 μg/kg/day intraperitoneally; Peprotech, Rocky Hill, NJ) group (*n* = 12), and Cis + G-CSF + AMD3100 (5 mg/kg subcutaneously; Sigma-Aldrich, St. Louis, MO) group (*n* = 12). G-CSF treatment was administered over five consecutive days, and AMD3100 was administered 60 min before AKI induction. At 96 h after the last cisplatin injection, the mice were euthanized. Blood and tissue samples were collected to assess renal function and tissue damage.

### Ablation of bone marrow stem cells in mice

To confirm bone marrow stem cell mobilization following G-CSF/AMD3100 administration, mice were irradiated with 2 separate doses of 4.5 Gy of whole-body γ-radiation at a 2-h interval to induce bone marrow ablation (BMA). Mice were randomly assigned to the following groups: the irradiation + saline group, irradiation + G-CSF/AMD3100 group, non-irradiation + saline group, and non-irradiation + G-CSF/AMD3100 group. Beginning 1 day after irradiation, G-CSF/AMD3100 was administered as described in the above-mentioned treatment protocol. Control mice that were not subjected to irradiation were injected with an equivalent dose of saline (*n* = 6 per group). The mice were euthanized 96 h after treatment, and peripheral blood samples were collected for subsequent flow cytometry (FCM) analyses.

### FCM analysis of the peripheral blood

Mice were anaesthetized with chloraldurat, and peripheral blood samples were collected for FCM analyses. Red blood cells were lysed using Lysing Buffer (Sigma). The remaining cells were labelled with FITC-labelled rat anti-mouse CD34 (diluted 1:100; BD Biosciences, #560238), APC-labelled rat anti-mouse CD133 (diluted 1:100; BioLegend, #141207), FITC-labelled rat anti-mouse CD44 (diluted 1:100; BD Biosciences, #553133), PE-labelled rat anti-mouse CXCR4 (diluted 1:200; BD Biosciences, #561734), and PE-labelled rat anti-mouse C-kit (diluted 1:100; BD Biosciences, #553355).

### Measurement of blood urea nitrogen (BUN) and serum creatinine levels

Serum samples were obtained from mice 96 h after cisplatin injection. BUN and creatinine levels were subsequently measured using an autoanalyser (Hitachi 7150 Auto-analyzer; Hitachi, Tokyo, Japan).

### Tissue processing and histopathological scoring

After the mice were euthanized, kidney specimens were fixed immediately in 10% buffered formalin for 24 h and then embedded in paraffin, and some kidney specimens were embedded in OCT for freezing. The kidney tissues were cut into 3-μm-thick sections for periodic acid-Schiff staining (PAS) and immunohistochemistry (IHC). Tubular injury was diagnosed based on the presence of tubular epithelial necrosis, cast formation, tubular dilatation, and brush border loss. Renal injury severity was scored in a blinded fashion as described in a previous study based on the percentage of tubule lesions in ten randomly selected [18] non-overlapping fields (magnification, × 200) as follows: 0, 0%; 1, ≤10%; 2, 11–25%; 3, 26–45%; 4, 46–75%; and 5, 76–100%.

### Immunofluorescence and IHC

IHC was performed to identify Ki-67-positive cells, which were counted in 20 randomly selected cortical and outer medullary (OSOM) fields at a magnification of × 400 by a blinded investigator. In addition, kidney tissue sections were subjected to immunofluorescence staining for BrdU using mouse anti-BrdU monoclonal antibodies (Roche) and DyLight 594-conjugated secondary antibodies (Amyjet) to evaluate tubular epithelial cell proliferation. The number of BrdU-positive cells was determined by counting the number of positive nuclei in 20 randomly selected non-overlapping cortical and OSOM fields at a magnification of × 400.

### Apoptosis assay

Apoptosis was evaluated using a terminal deoxynucleotidyl transferase dUTP nick end-labelling (TUNEL) assay kit (Roche, Indianapolis, IN, USA). Briefly, C57BL/6J mouse kidney sections were deparaffinized, rehydrated, digested with proteinase K, and labelled with TUNEL reaction mixture for 60 min at 37 °C. The TUNEL-positive cells, which corresponded to apoptotic tubular epithelial cells, were counted in 20 randomly selected cortical fields at high-power magnification (× 400). All tissue sections were viewed and labelled by a blinded examiner.

### Western blot analysis

A total of 50 μg of total renal cell lysate was separated by SDS-PAGE and transferred onto polyvinylidene difluoride (PVDF) membranes (Millipore; Billerica, MA, USA). Western blotting was performed as described in our previous study [19]. The following primary antibodies were used: PCNA (diluted 1:500, Proteintech, USA, # 60097-1-Ig), Bcl-2 (diluted 1:1000, Santa Cruz, #sc-7382), and Bax (diluted 1:1000, Santa Cruz, #sc-7480). HRP-conjugated anti-mouse and anti-rabbit secondary antibodies (diluted 1:3000; Amyjet, #AS09-602 and AS10-1427) were used to detect proteins using an ECL Assay Kit (Bipec Biopharma). β-Tubulin (diluted 1:1000; Proteintech, USA, # 10094-1-AP) or β-actin (diluted 1:1000; Proteintech, USA, #20536-1-AP) was used as an internal control. Band intensity was quantified using the ImageJ software (1.44 P).

### Real-time reverse transcription-polymerase chain reaction (PCR) analysis

Total RNA was extracted from tissues with TRIzol Reagent (TaKaRa) according to the manufacturer’s instructions. RNA reverse transcription was conducted using a PrimeScript™ RT Reagent Kit (TaKaRa). PCR enzymes and master mixes (DBI Bioscience), along with primers specific for mouse GAPDH, TNF-α, IL-6, IL-10, kidney injury molecule-1 (Kim1), and neutrophil gelatinase-associated lipocalin (Ngal), were used for real-time PCR, Kim1, and Ngal. Relative expression levels were normalized to the levels of GAPDH and calculated using the 2^−ΔΔCt^ method, where △△CT = (Ct,_Target gene_ − Ct,_GAPDH_)_Target group_ − (Ct,_Target gene_ − Ct,_GAPDH_)_reference group_ [20]. The primer sequences were as follows:

IL-6 forward (F): 5′-TCCAGTTGCCTTCTTGGGAC-3′; reverse (R): 5′-TGCACAACTCTTTTCTCATTTCCAC-3′

IL-10 forward (F): 5′-ATCAGCAGGGGCCAGTAC-3′; reverse (R): 5′-AAGGCTTGGCAACCCAAGT-3′

Kim1 forward (F): 5′-TACCTGGAGTAATCACACTGAAGCA-3′; reverse (R): 5′-TTCAATCTTAGAGACACGGAAGGC-3′

Ngal forward (F): 5′-GGCAGCTTTACGATGTACAGCA-3′; reverse (R): 5′-TCTGATCCAGTAGCGACAGCC-3′

TNF-α forward (F): 5′-TCACAAAACTTGAGAGTCGTGGTG-3′; reverse (R): 5′-AAAGTGGCTCTACGTTATATTCTGCC-3′

GAPDH forward (F): 5′-GCCAGCCTCGTCTCATAGACA-3′; reverse (R): 5′-AGAGAAGGCAGCCCTGGTAAC-3′

### Statistical analysis

The results are presented as the mean ± SEM. Multiple group comparisons were performed by ANOVA followed by Bonferroni multiple-comparison post hoc test. For survival analysis, the Kaplan-Meier method was used, and the groups were compared using the log-rank test. Statistical analysis was carried out using the SAS statistical software for Windows version 8.2 (SAS Institute, Cary, NC). All tests were two-tailed, and a *P* value < 0.05 was considered significant.

## Results

### Pretreatment with the combination of G-CSF and AMD3100 improves the survival of cisplatin-injected mice

**Fig. 2:**
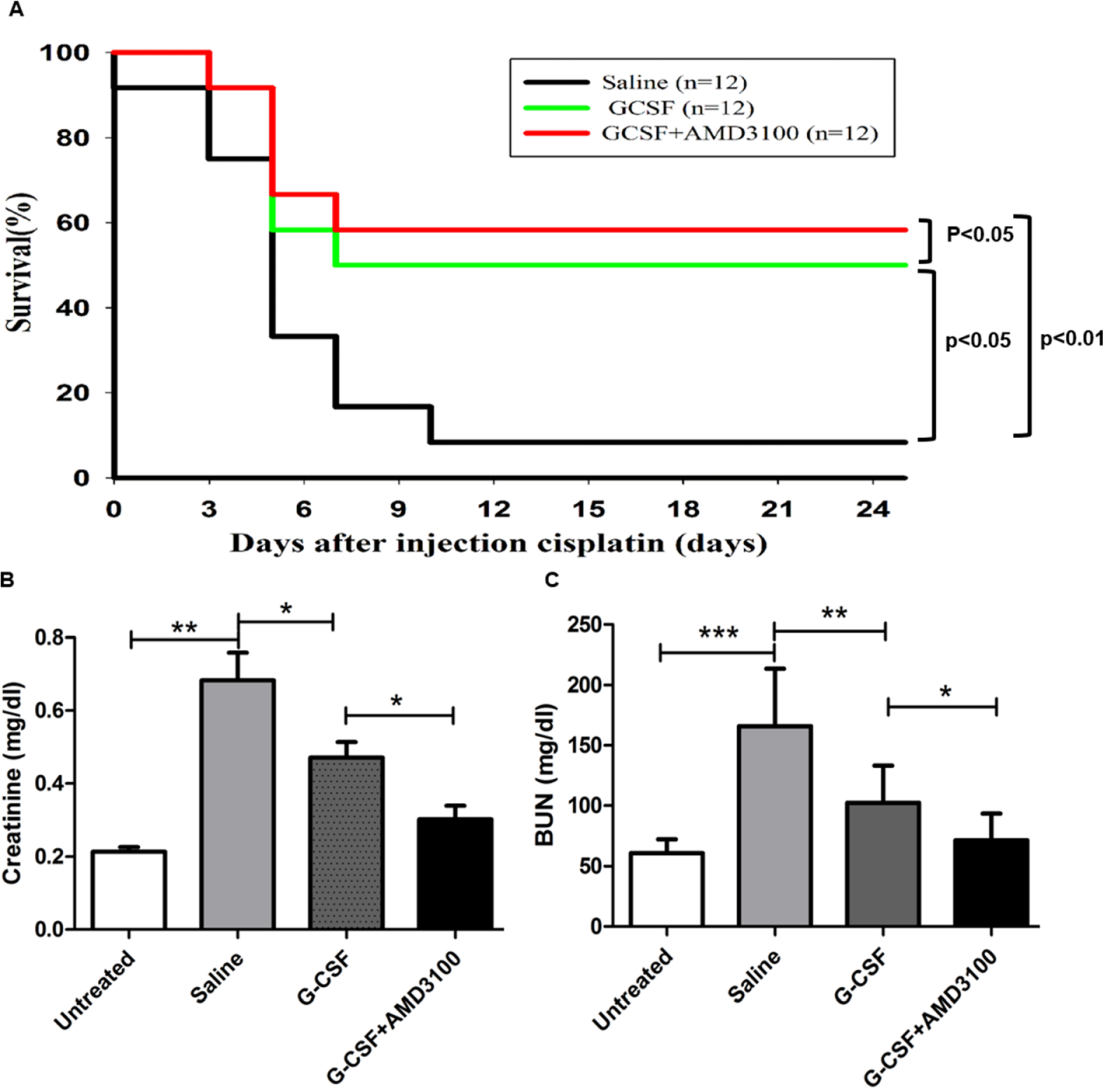
Survival rates and blood of serum creatinine and urea nitrogen (BUN) levels of cisplatin-administered mice that were pretreated with or without G-CSF and/or AMD3100. **a** C57BL/6 mice received an injection of G-CSF (200 μg/kg/day), G-CSF (200 μg/kg/day)/AMD3100 (5 mg/kg), or saline (as control) into the intraperitoneal before treated with cisplatin. Mice were observed to determine the survival rate (*n* = 12, per group). **b** Serum creatinine and **c** serum BUN levels of the mice 4 days after cisplatin injection are shown. “Untreated” means mice without treatment with cytokine and cisplatin. Results are expressed as mean ± SD. *P < 0.05, **P < 0.01 and *** *P* < 0.001

BMSCs can repair injured renal tubules, and G-CSF can mobilize BMSCs into the peripheral blood. Therefore, we examined the ability of pretreatment with G-CSF/AMD3100 to prolong the survival of mice injected with cisplatin. As shown in Fig. 2a, only 16.7% of mice survived up to 10 days after cisplatin injection, whereas 58.3% of mice pretreated with G-CSF/AMD3100 survived up to 10 days after cisplatin injection (*P* < 0.01). Furthermore, the G-CSF/AMD3100 group had a better survival rate than the G-CSF group (*P* < 0.05). These results suggest that combinatorial G-CSF/AMD3100 therapy has the ability to protect against cisplatin-induced renal toxicity.

### The combination of G-CSF/AMD3100 ameliorates cisplatin-induced renal functional deterioration

We measured BUN and serum creatinine levels to assess the severity of renal dysfunction in each group after cisplatin injection. As shown in Fig. 2b, c, 96 h after cisplatin injection, serum creatinine and BUN levels were significantly increased in the saline pretreatment group compared with the control group (*P* < 0.01). The mice that received G-CSF or G-CSF/AMD3100 exhibited significantly lower serum creatinine and BUN levels than mice that were treated with saline. Moreover, the levels of BUN and creatinine were notably lower in the G-CSF/AMD3100 pretreatment group than in the G-CSF-treated alone group (*P* < 0.05). These results suggest that pretreatment with G-CSF/AMD3100 significantly attenuates the deterioration of renal function after cisplatin-induced AKI and that the combination of G-CSF/AMD3100 is more effective than pretreatment with G-CSF alone.

### The combination of G-CSF and AMD3100 ameliorates renal tubule lesions

**Fig. 3:**
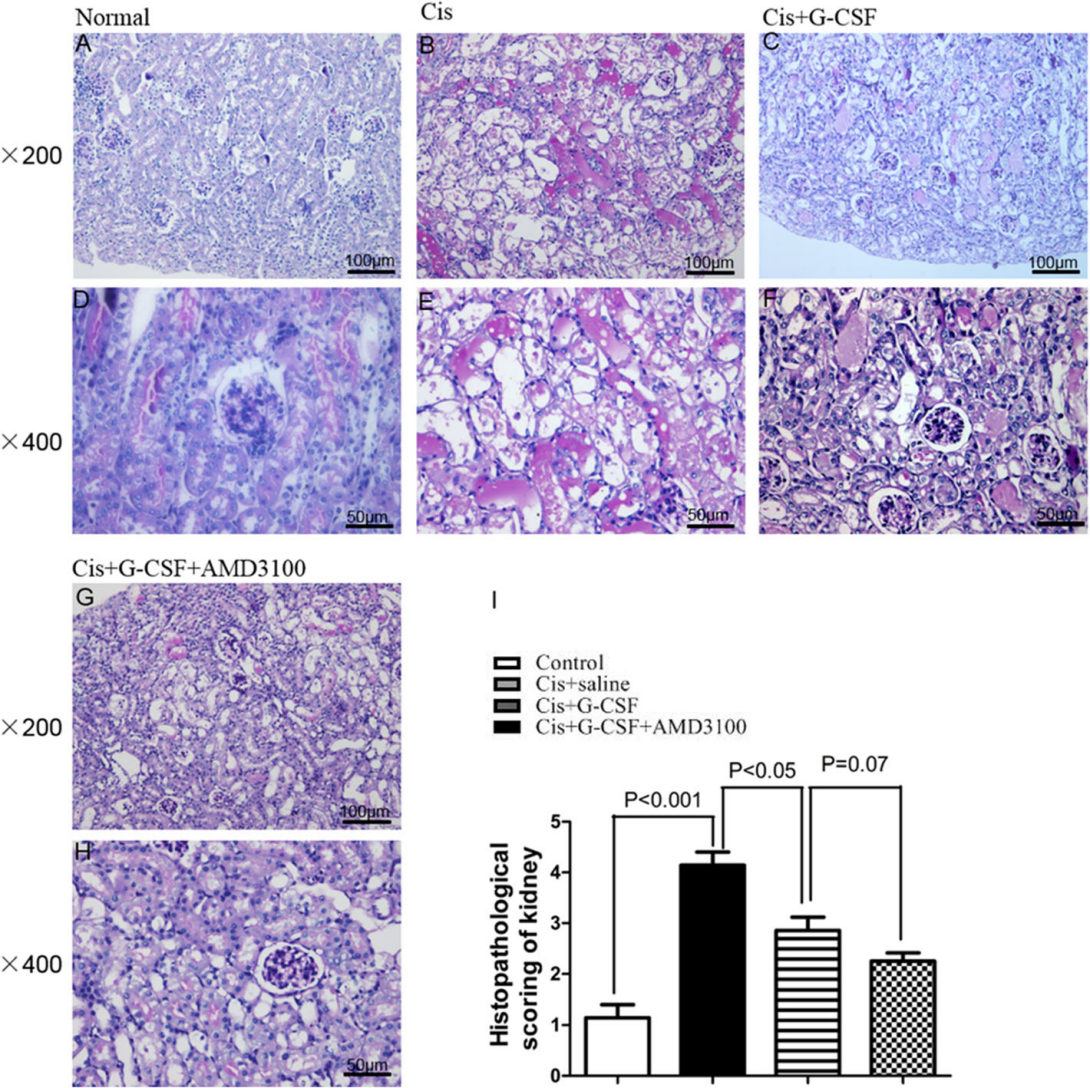
Histologic analyses of kidneys of cisplatin-treated mice. C57BL/6 mice received an injection of G-CSF (200 μg/kg per d), G-CSF (200 μg/kg per d)/AMD3100 (5 mg/kg), or saline (as a control of cytokines) into the intraperitoneal space. The day after the last injection of cytokines, single intraperitoneal injections of cisplatin (20 mg/kg body wt) were given to these mice. Morphologic changes in PAS staining are shown. The figures indicated the renal tubules of saline-pretreated mice (**a**, **d**), cisplatin + saline-treated mice (**b**, **e**), cisplatin + G-CSF-pretreated mice (**c**, **f**), and cisplatin + G-CSF/AMD3100-pretreated mice (**g**, **h**). Representative data are shown for three independent experiments. Magnification, × 200 in **a**–**c** and **g** and × 400 in **d**–**f** and **h**. Histopathological scoring of cisplatin-induced renal injury (**i**). Tubular injury was defined as tubular necrosis, cast formation, loss of brush border in the renal tubules, and tubular dilatation. Data are presented as the means ± SD (*n* = 9 in each group)

We next examined the histological changes in the kidney after treatment with cisplatin. Renal tissues were subjected to PAS staining (Fig. 3a–h). According to histological scoring, saline-treated mice exhibited more severe tissue injury than G-CSF- and G-CSF/AMD3100-treated mice, and G-CSF/AMD3100-treated mice exhibited significantly lower histopathological scores than G-CSF-treated mice after cisplatin injection (*P* < 0.05; Fig. 3i). These results suggest that G-CSF/AMD3100 pretreatment protects against cisplatin-induced renal damage.

### The combination of G-CSF and AMD3100 mobilizes BMSCs

**Fig. 4:**
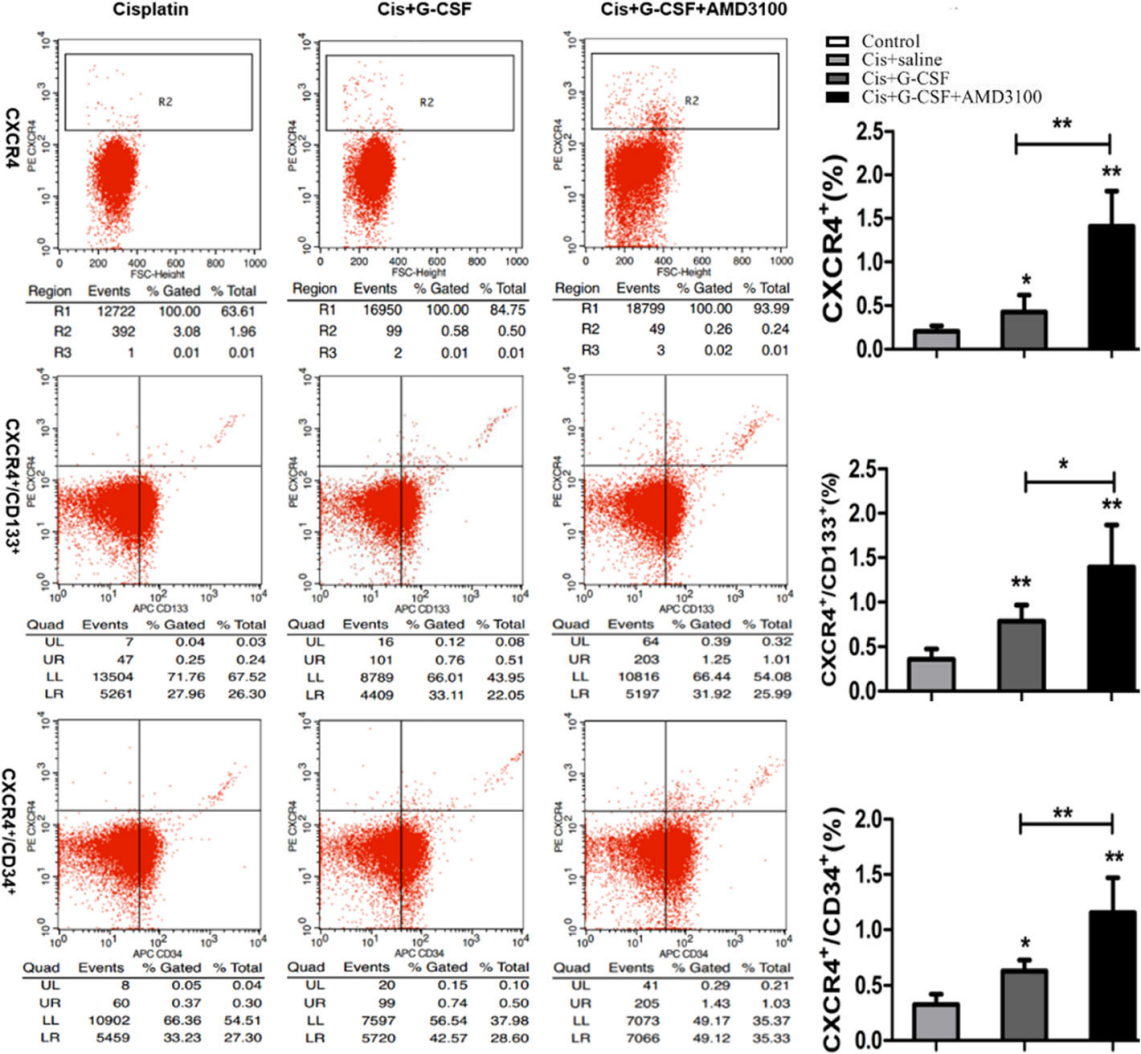
Effects of G-CSF/AMD3100 on the mobilization of stem cells. Four days after cisplatin injection, the peripheral blood of the mice was obtained and analysed by a flow cytometer. **a** CXCR4^+^ cells. **b** CXCR4^+^CD34^+^ cells. **c** CXCR4^+^CD133^+^ cells. Data represent the mean ± SD (*n* = 6 per group). *P < 0.05, **P < 0.01, and vs. the cisplatin group and the indicated test group

CD34^+^ and CD133^+^ cells are widely recognized as haematopoietic stem cell (HSC) and endothelial progenitor cell (EPC) biomarkers, respectively. In this study, BMSCs in peripheral blood samples from C57BL/6 male mice were analysed by FCM analysis. As expected, compared with saline treatment, G-CSF and G-CSF/AMD3100 treatment significantly enhanced circulating CXCR4^+^ cells, as shown in Fig. 4 (*P < 0.05 and **P < 0.01, respectively). In addition, there were more CXCR4^+^ cells in the G-CSF/AMD3100 group than in the G-CSF-treated group (*P* < 0.01) (Fig. 4a). CXCR4^+^CD34^+^ and CXCR4^+^CD133^+^ cells were harvested and identified by double-staining (Fig. 4b, c). Compared to G-CSF treatment, G-CSF/AMD3100 treatment increased the numbers of CXCR4^+^CD34^+^ and CXCR4^+^CD133^+^ cells in the peripheral blood by 1.8-fold and 1.9-fold, respectively. The percentages of CXCR4^+^CD34^+^ and CXCR4^+^CD133^+^ cells were markedly higher in the G-CSF/AMD3100-treated and G-CSF-treated groups than in the saline-treated group. We also measured the numbers of CXCR4^+^CD44^+^ and C-kit^+^ peripheral blood mononuclear cells, which exhibited the same trend as CXCR4^+^CD34^+^ and CXCR4^+^CD133^+^ (Supplemental Fig. 1). These findings suggest that G-CSF/AMD3100 effectively mobilizes stem cells into the peripheral blood to facilitate tubule repair and regeneration.

### BMA prevents BMSC mobilization induced by the combination of G-CSF and AMD3100

To further determine whether G-CSF/AMD3100 administration mobilizes BMSCs into the peripheral blood, we induced BMA via irradiation to prevent BMSC development and mobilization. As shown in Supplemental Fig. 2, the percentages of CXCR4^+^CD34^+^ and CXCR4^+^CD133^+^ cells in the bone marrow were not different between the irradiation + saline group and irradiation + G-CSF/AMD3100 group (*P* > 0.05); however, the percentages of these cells were significantly higher in the non-irradiation + G-CSF/AMD3100 group than in the non-irradiation + saline and irradiation + G-CSF/AMD3100 groups (*P* < 0.01). These results further prove that BMSCs are mobilized into the peripheral blood by G-CSF/AMD3100.

### The combination of G-CSF and AMD3100 enhances tubular epithelial cell regeneration

**Fig. 5:**
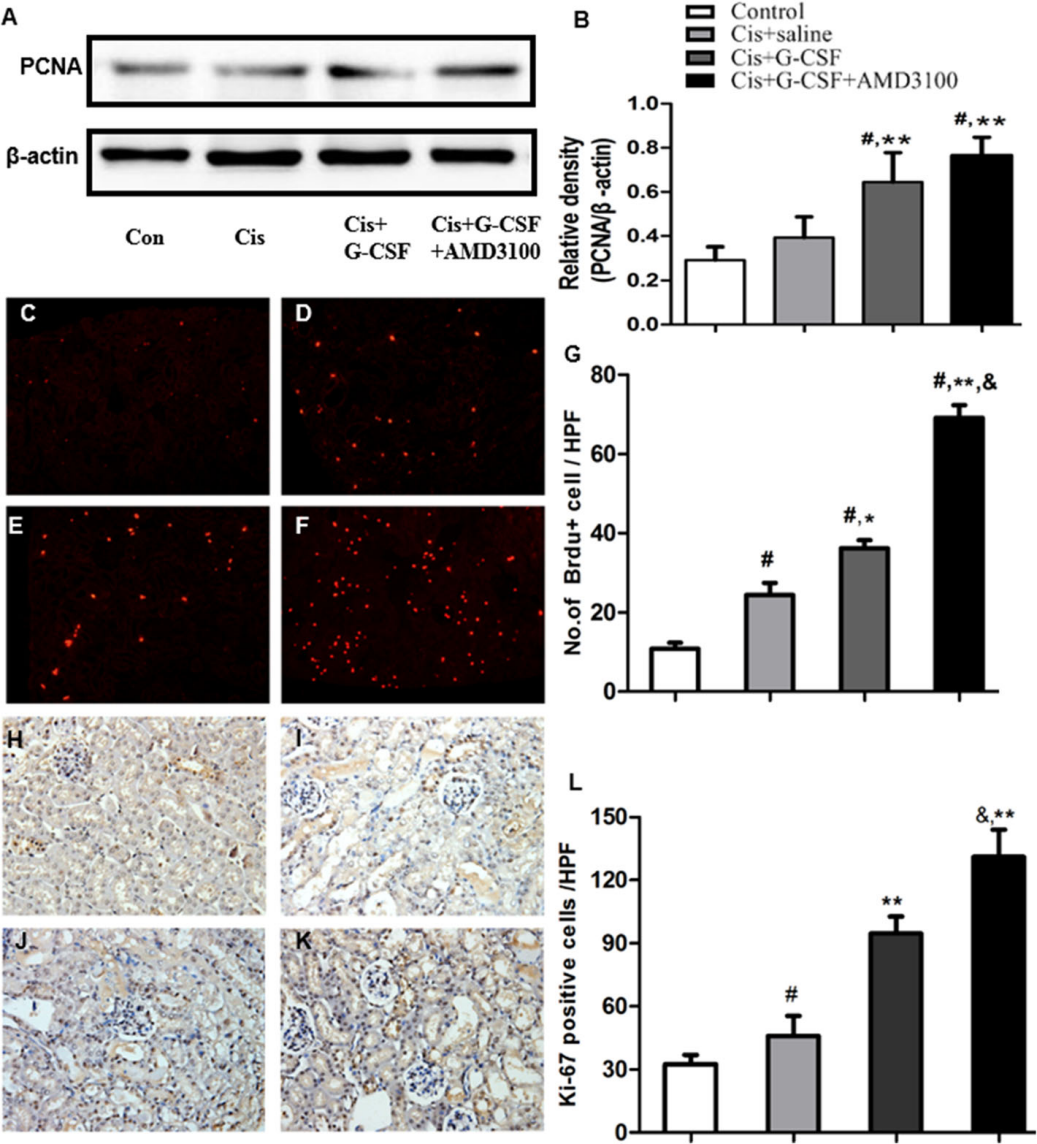
Effects of G-CSF/AMD3100 on renal tubular regeneration. **a**, **b** Western blot analyses of PCNA protein. Notably higher PCNA protein expression in the G-CSF/AMD3100 pretreatment group than in the saline or G-CSF-treated group. **c**–**f** Immunofluorescent staining tested the expression of Brdu in renal tubular, and red fluorescence represents the nucleus of Brdu^+^ cells. **g** The number of Brdu^+^ cells/HPF. The figures indicate Brdu^+^ cells in the renal tubules of saline-treated mice (control) (**c**), cisplatin + saline-treated mice (**d**), cisplatin + G-CSF-pretreated mice (**e**), and cisplatin + G-CSF/AMD3100 pretreated mice (**f**). **h**–**k** Immunohistochemical staining tested the expression of Ki-67 in renal tubular. **l** The number of Ki-67^+^ cells/HPF. The figures indicate Ki-67^+^ cells in the renal tubules of saline-treated mice (control) (**h**), cisplatin-treated mice (**i**), cisplatin + G-CSF-pretreated mice (**j**), and cisplatin + G-CSF/AMD3100-pretreated mice (**k**). Data represent the mean ± SD (*n* = 6 per group). *P < 0.05, **P < 0.01, and compared with the cisplatin group; ^#^*P* < 0.05 compared with the control group; ^&^*P* < 0.05 compared with the G-CSF group. Original magnification × 400

To determine whether G-CSF/AMD3100 pretreatment facilitates tubular epithelial cell proliferation and regeneration, we examined the protein expression of PCNA in the kidney after cisplatin injection. The Western blotting analysis revealed that PCNA expression was significantly increased in the G-CSF/AMD3100-treated mice compared with the saline-treated mice (*P* < 0.05). G-CSF/AMD3100-treated mice exhibited higher protein levels of PCNA than G-CSF-treated mice (Fig. 5a, b). We also assessed the effects of G-CSF/AMD3100 treatment on tubular cell proliferation and regeneration by counting the number of BrdU^+^ cells in each group. G-CSF-treated and G-CSF/AMD3100-treated mice exhibited a higher number of BrdU^+^ cells than saline-treated mice, and G-CSF/AMD3100-treated mice exhibited a significantly higher number of BrdU^+^ cells than G-CSF-treated mice (69.1 ± 8.3 cells/HPF in the G-CSF group and 36.1 ± 5.5 cells/HPF in the G-CSF/AMD3100 treated group; *P <* 0.05; Fig. 5c–g). Similar results were also observed for Ki-67 expression. A low number of Ki-67-positive cells were detected in renal tissues in control and saline-treated mice (32.4 ± 4.4 cells/HPF in the control group and 45.9 ± 5.7 cells/HPF in the control group) (Fig. 5h, i). G-CSF/AMD3100 treatment resulted in a 4-fold increase in the number of Ki-67 cells compared with saline treatment (*P <* 0.01) and a 1.5-fold increase compared with G-CSF treatment (Fig. 5h–l). These results suggest that G-CSF/AMD3100 pretreatment promotes tubular epithelial cell regeneration to a greater extent than G-CSF pretreatment.

### The combination of G-CSF and AMD3100 promotes the protective effect of BMSCs against cisplatin-induced injury

**Fig. 6:**
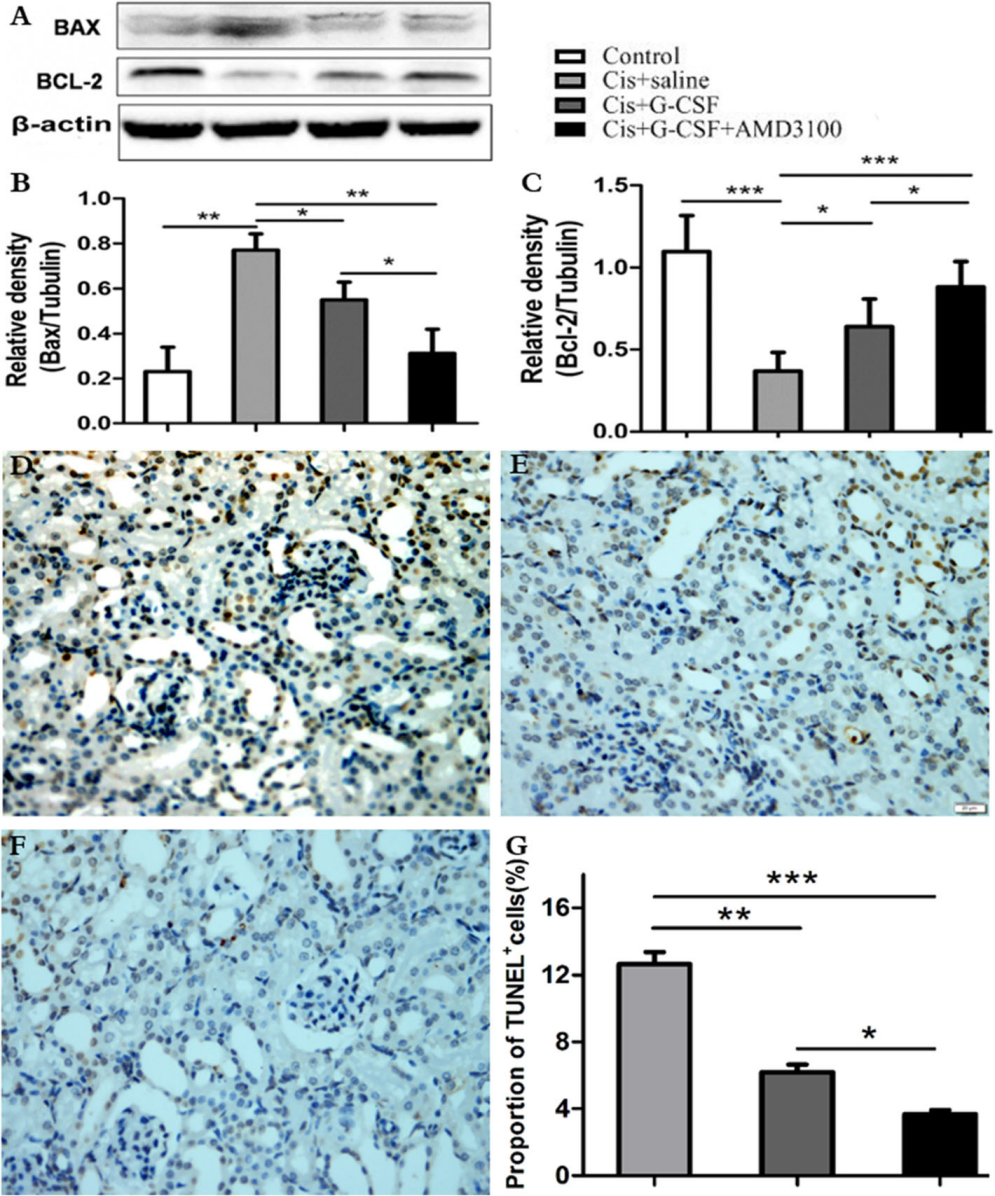
Effects of G-CSF/AMD3100 on renal tubular cell apoptosis. **a**–**c** Western blot analyses of BCL-2 and BAX protein. G-CSF/AMD3100 pretreatment showed a significant increase in BCL-2 expression but a decrease in BAX expression. **d**–**g** Representative micrographs showing the TUNEL immunostaining amongst different groups. **d** Cisplatin + saline-treated mice. **e** Cisplatin + G-CSF-pretreated mice. **f** Cisplatin + G-CSF/AMD3100-pretreated mice. **g** Quantitative analysis for the number of TUNEL staining-positive cells amongst different groups. Data represent the mean ± SD (*n* = 6 per group). *P < 0.05, **P < 0.01, ***P < 0.001. Original magnification × 400

To determine the relationship between cisplatin nephrotoxicity and apoptosis, we performed Western blotting to quantify Bcl-2 and Bax protein expression. Bcl-2 expression was notably lower in the saline-treated group than in the other three groups and was significantly lower in the G-CSF-treated group than in the G-CSF/AMD3100-treated group (*P* < 0.05, Fig. 6a, c). In contrast, the level of Bax expression was notably higher in the saline-treated group than in the G-CSF and G-CSF/AMD3100 groups and significantly higher in the G-CSF-treated group than in the G-CSF/AMD3100 group (*P* < 0.05; Fig. 6a, b). Furthermore, to evaluate renal tubular epithelial cell (RTEC) damage, we determined the percentages of apoptotic RTECs via the TUNEL assay. Compared with saline-treated mice, G-CSF-treated mice and G-CSF/AMD3100-treated mice exhibited a significantly decreased proportion of TUNEL^+^ cells following the induction of nephrotoxicity (*P* < 0.01 and *P* < 0.001, respectively). Interestingly, G-CSF/AMD3100-treated mice exhibited significantly fewer TUNEL^+^ cells than G-CSF-treated mice (Fig. 6d–g). In addition, the qRT-PCR analysis revealed that the levels of Kim-1 in G-CSF/AMD3100-treated mice were 2-fold lower than those in saline-treated mice and that the level of Ngal in the G-CSF/AMD3100-treated group was 1.9-fold lower than that in saline-treated mice. G-CSF/AMD3100 treatment also decreased Kim1 and Ngal mRNA expression to a greater extent than G-CSF administration alone in the AKI model, although these differences were not statistically significant (*P* = 0.065 and *P* = 0.058, respectively) (Supplemental Fig. 3). These results indicate that G-CSF/AMD3100 administration prevents cisplatin-induced RTEC damage.

### Impacts of the combination of G-CSF and AMD3100 on changes in inflammation after nephrotoxic injury

**Fig. 7:**
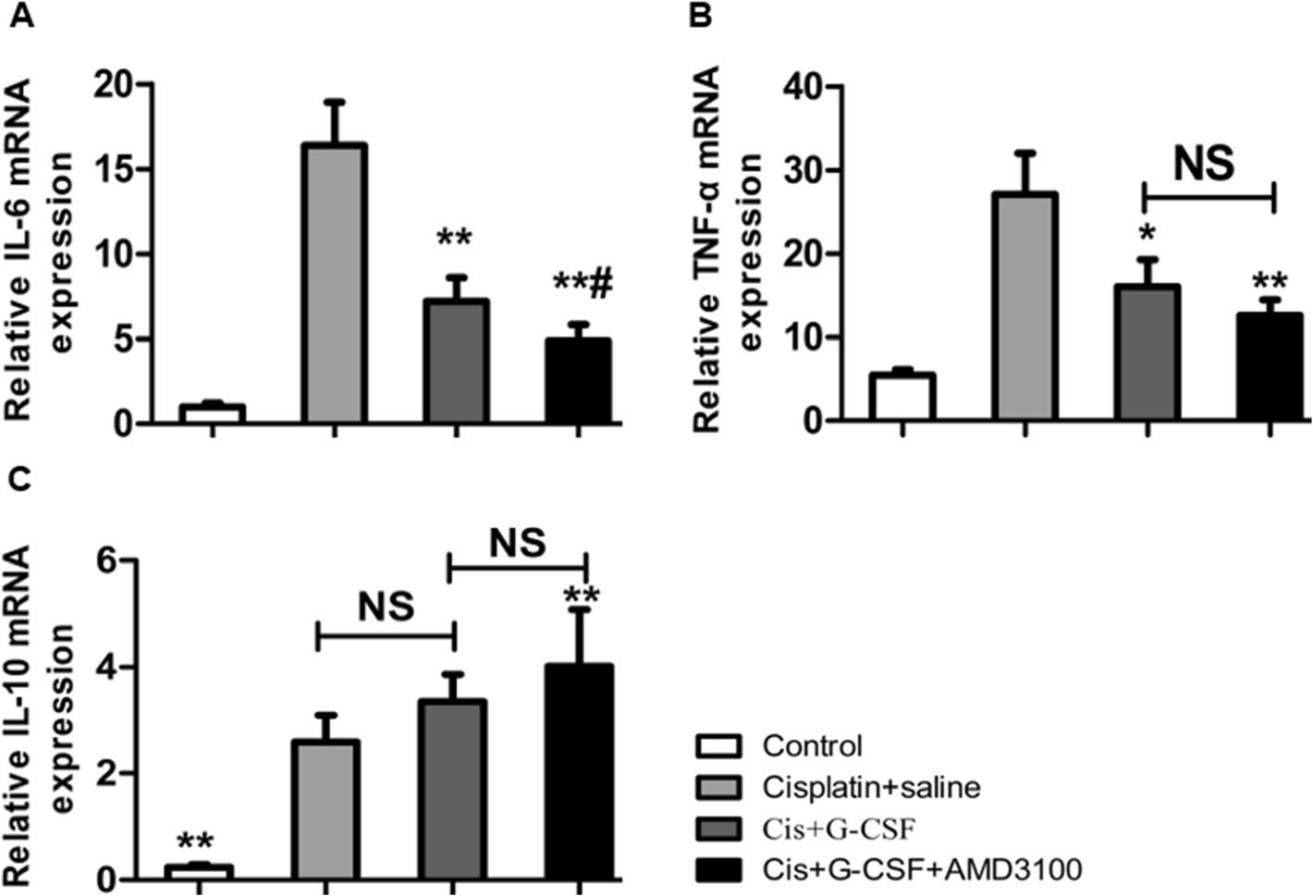
Effects of G-CSF/AMD3100 on the inflammatory cytokine mRNA expression. Total RNA was extracted from renal tissue in the saline-, cisplatin + saline-, G-CSF-, and G-CSF/AMD3100-treated mice, 4 days after AKI and analysed for IL-6 (**a**), TNF-α (**b**), and IL-10 (**c**) mRNA expression by real-time RT-PCR analysis. The results are expressed as the means ± SD (*n =* 6).*P < 0.05, **P < 0.01, vs. the cisplatin group and the indicated test group

To investigate whether G-CSF/AMD3100 affects the levels of cytokines in mice with cisplatin-induced nephrotoxicity, we examined the expression of the proinflammatory cytokine IL-6, which increases abruptly in cisplatin-induced nephrotoxicity. G-CSF/AMD3100 treatment significantly decreased IL-6 mRNA expression (*P* < 0.01; Fig. 7a). We also observed similar changes in TNF-α levels, although no significant difference in the levels of TNF-α was observed between the G-CSF- and G-CSF/AMD3100-treated mice (*P* = 0.065; Fig. 7b). However, mice with cisplatin-induced nephrotoxicity exhibited much higher mRNA expression levels of the anti-inflammatory cytokine IL-10 than control mice. Moreover, compared with saline administration, G-CSF/AMD3100 administration caused a significant increase in the mRNA levels of IL-10 mRNA (*P* < 0.01; Fig. 7c). Taken together, these data indicate that G-CSF/AMD3100 treatment contributes to further inhibiting the inflammatory reaction in this experimental setting.

## Discussion

In this study, we explored the effects of the combination of G-CSF and AMD3100 in a mouse model of cisplatin-induced AKI. The major findings of this study are as follows: first, combinatorial G-CSF/AMD3100 therapy significantly protects against tubular epithelial cell apoptosis and tubular damage, attenuates the deterioration of renal function, and improves the survival of mice after cisplatin-induced nephrotoxicity. Second, combinatorial G-CSF/AMD3100 more efficiently mobilizes and releases BMSCs into peripheral circulation than either treatment alone. Third, combinatorial G-CSF/AMD3100 therapy exerts anti-inflammatory effects with no obvious side effects.

Accumulating evidence has demonstrated that stem cells, especially BMSCs, mobilize to sites of renal injury to facilitate tissue repair and regeneration [21–23]. These studies suggest the therapeutic potential of bone marrow stem cell application for the treatment of kidney diseases. However, how to efficiently mobilize stem cells into the peripheral blood to restore renal function after AKI is not fully clear. G-CSF treatment was shown to attenuate renal dysfunction and infarction after acute ischaemia/reperfusion and ameliorate toxic renal injury caused by cisplatin in mice [7, 22]. A previous study by Ikehara et al. reported that M-CSF enhances the effects of G-CSF on mobilizing bone marrow cells to accelerate improvements in renal functions and prevent renal tubular injury induced by cisplatin [22]. However, this study found that there was no significant difference in the survival rate between the group pretreated with M-CSF + G-CSF and the group pretreated with G-CSF only. Here, we showed that the mice pretreated with the combination of G-CSF and AMD3100 had a better survival rate than mice pretreated with G-CSF only after cisplatin injection. This beneficial effect is related to the special function of AMD3100, which inhibits the homing of stem cells to bone marrow niches and synergistically augments G-CSF-induced mobilization of HSCs [9], thus promoting the migration of more HSCs into injured tissue (Supplemental Fig. 4). Our experimental results showed that there were more CXCR4^+^CD34^+^ and CXCR4^+^CD133^+^ cells in the peripheral blood in G-CSF/AMD3100-treated mice than in G-CSF-treated mice. These results, to some extent, support theoretical speculation. These findings are consistent with those of previous studies demonstrating that the combination of G-CSF/AMD3100 mobilizes BMSCs more effectively than G-CSF alone [24–26]. Moreover, to show that the stem cells mobilized by G-CSF/AMD3100 were derived from the bone marrow, we induced BMA by irradiation. The percentages of CXCR4^+^CD34^+^ and CXCR4^+^CD133^+^ cells were not different between the irradiation + saline group and irradiation + G-CSF/AMD3100 group. The results may indirectly indicate that G-CSF and AMD3100A have no effect on stem cell division or that the combination of G-CSF and AMD3100 has no effect on cell number.

AMD3100 was approved by the United States Food and Drug Administration for the autologous transplantation of bone marrow cells in patients with non-Hodgkin’s lymphoma in December 2008 [27]. After more than 10 years of clinical application for non-Hodgkin’s lymphoma, ovarian cancer, and acute ischaemic stroke, it has been observed to be safe in patients. A previous study suggested that AMD3100 has the ability to mobilize HSCs into the circulation in lymphoma and multiple myeloma patients for whom HSC mobilization therapy with G-CSF alone failed [27, 28]. Several studies have demonstrated that acute application of AMD3100 enhances tissue repair [11, 29], whereas continuous AMD3100 administration has adverse effects on tissue regeneration [14, 15]. When AMD3100 is administered after 4 to 5 days of G-CSF administration, the number of circulating CD34 cells significantly increases [30]. AMD3100 exerts rapid cell mobilization effects that peak within 1–3 h of administration in mice [26, 31]. Therefore, we selected a treatment regimen in which ADM3100 was administered subcutaneously after G-CSF treatment five consecutive days and 1 h before cisplatin injection. The results of this study suggest that this treatment regimen may have positive effects on stem cell mobilization without exerting negative effects on stem cell homing.

Previous studies have demonstrated that the mechanism underlying stem cell-based therapy includes angiogenesis, stem cell homing, anti-inflammatory reactions, antioxidative stress, immunomodulation, and transdifferentiation [32, 33]. In fact, the mechanisms underlying the improvements in organ function following BMSC therapy could be even more complex. Zuk et al. [15]. reported that AMD3100 administration ameliorates I/R-induced kidney injury, as evidenced by reduced leukocyte infiltration and proinflammatory chemokine/cytokine expression, rather than exerting a bone marrow HSC-mediated effect. AMD3100 also mobilizes murine long-term repopulating cells engrafted in primary and secondary lethally irradiated mice [26]. We found that pretreatment with G-CSF/AMD3100 or G-CSF in cisplatin-treated mice can contribute to promoting renal repair by promoting cell proliferation and decreasing apoptosis, as indicated by increased protein expression levels of several cell proliferation-related markers, such as PCNA, Ki-67 and BrdU. In addition, significantly decreased protein expression levels of Bax and notably enhanced protein expression levels of Bcl-2 were observed in cisplatin-treated mice pretreated with G-CSF/AMD3100. Furthermore, these effects were greater in mice pretreated with G-CSF/AMD3100 than in those pretreated with G-CSF alone. Taken together, our findings are consistent with those of previous studies demonstrating that mesenchymal stem cell (MSC) treatment can alleviate experimental cisplatin-induced nephrotoxicity, at least in part by attenuating apoptosis and tubular injury and by promoting tubular regeneration [15, 34–36]. However, we failed to elucidate the mechanism by which BMSCs transdifferentiate into RTECs, which is one of the limitations of this study. Therefore, further investigations of the exact mechanisms involved in the effect of BMSC therapy against renal injury are warranted.

Numerous studies have demonstrated that BMSCs exert potent immunomodulatory effects in vitro and in vivo [37–39]. In the current study, we observed that the mRNA expression levels of IL-10 were higher in cisplatin-treated animals pretreated with G-CSF/AMD3100 than in animals pretreated with G-CSF alone. Milwid et al. [38]. reported that BMSC-secreted IL-10 contributes to the attenuation of severe cisplatin-induced AKI. Moreover, the levels of the proinflammatory cytokines IL-1β, IL-6, and TNF-α are increased in renal tubular cells in the context of cisplatin-induced AKI [40, 41]. The IL-6 and TNF-α mRNA expression levels observed in this study are consistent with these findings. We observed significantly lower levels of IL-6 and TNF-α following G-CSF/AMD3100 or G-CSF pretreatment, which is consistent with the results of Overath et al. [42], who observed that adipose-derived MSCs pretreated with exposure to hypoxia significantly decreased proinflammatory cytokine levels and significantly attenuated cisplatin-induced renal injury in mice.

## Conclusions

Our results suggest that compared to the treatment with G-CSF alone, the combination of G-CSF and AMD3100 can significantly enhance the mobilization and homing of BMSCs to sites of renal tissue damage, alleviate renal tissue injury, and improve renal function recovery after cisplatin-induced AKI. Taken together, our findings provide some evidence for the potential of the combination of G-CSF and AMD3100 as a novel therapeutic strategy for cisplatin-induced nephrotoxicity.

## Supplementary Information

**Additional file 1 **: **Supplemental Figure 1.** Effects of G-CSF and/or AMD3100 on mobilization of stem cells. The percentage of c-kit^+^ cells (A) and CXCR4^+^/CD44^+^ cells in the circulating blood was performed flow cytometry analysis after induction of AKI in control cisplatin, G-CSF and G-CSF/AMD treated mice. Results are expressed as the means ± SD (*n=*5 per group),*P<0.05, **P<0.01, vs. the cisplatin group, and the indicated test group. **Supplemental Figure 2.** Effect of bone marrow ablation (BMA) and G-CSF/AMD3100 treatment in C57BL/6J mice on mobilization of stem cells in the peripheral blood. Irradiated C57BL/6J mice received treatment with G-CSF/AMD3100 or saline, as described in the Materials and Methods section. 96 hours after the last injection of cytokines, collected of peripheral blood. The percentage of CXCR4^+^CD34^+^ cells (A), CXCR4^+^CD133^+^ cells (B) in the circulating blood was performed flow cytometry analysis. Results are expressed as the means ± SD (*n=*5 per group),*P<0.05, **P<0.01, NS : no significance. **Supplemental Figure 3.** Effects of G-CSF/AMD3100 on the Kim-1, Ngal mRNA expression. (A)The expression of Kim-1 mRNA was detected by RT-PCR. (B) The expression of Ngal mRNA was detected by RT-PCR. Results are expressed as the means ± SD (*n=*6), # *P*<0.001, vs. the control group; **P<0.01, vs. the cisplatin group and the indicated test group. **Supplemental Figure 4.** Schematic diagrams illustrating the mechanism of G-CSF/AMD3100 mobilizing bone marrow–derived stem cells rescues mice from Cisplatin-induced acute renal failure. Exogenous AMD3100 specifically blocks CXCR4 mediated SDF-1/CXCR4 interactions in bone marrow microenvironment, resulting in BMSCs snap out the bone marrow niche, mobilize to the peripheral blood, and homing into injured kidney. The BMSCs promote renal repair via improving renal tubular cells proliferation and regeneration, regulating apoptosis and inflammatory cytokines.

## Data Availability

All data generated or analysed during this study are included in this article.

## Abbreviations

BMSCs: Bone marrow-derived stem cells
AKI: Acute kidney injury
I/R: Ischaemia/reperfusion
G-CSF: Granulocyte colony-stimulating factor
BMA: Bone marrow ablation
FCM: Flow cytometry
BUN: Blood urea nitrogen
IHC: Immunohistochemical
TUNEL: Transferase dUTP nick end-labelling
PVDF: Polyvinylidene difluoride
PAS: Periodic acid-Schiff stain
HSC: Haematopoietic stem cell
EPC: Endothelial progenitor cell
RTEC: Renal tubular epithelial cell
