# Supplementary material for: The combination of G-CSF and AMD3100 mobilizes bone marrow-derived stem cells to protect against cisplatin-induced acute kidney injury in mice
Source: Stem Cell Res Ther. 2021 Mar 24;12:209. doi: 10.1186/s13287-021-02268-y (PMC7992860; doi:10.1186/s13287-021-02268-y)
Supplement: Supplementary file 1 — Additional file 1 : Supplemental Figure 1. Effects of G-CSF and/or AMD3100 on mobilization of stem cells. The percentage of c-kit+ cells (A) and CXCR4+/CD44+ cells in the circulating blood was performed flow cytometry analysis after induction of AKI in control cisplatin, G-CSF and G-CSF/AMD treated mice. Results are expressed as the means ± SD (n=5 per group),*P<0.05, **P<0.01, vs. the cisplatin group, and the indicated test group. Supplemental Figure 2. Effect of bone marrow ablation (BMA) and G-CSF/AMD3100 treatment in C57BL/6J mice on mobilization of stem cells in the peripheral blood. Irradiated C57BL/6J mice received treatment with G-CSF/AMD3100 or saline, as described in the Materials and Methods section. 96 hours after the last injection of cytokines, collected of peripheral blood. The percentage of CXCR4+CD34+ cells (A), CXCR4+CD133+ cells (B) in the circulating blood was performed flow cytometry analysis. Results are expressed as the means ± SD (n=5 per group),*P<0.05, **P<0.01, NS : no significance. Supplemental Figure 3. Effects of G-CSF/AMD3100 on the Kim-1, Ngal mRNA expression. (A)The expression of Kim-1 mRNA was detected by RT-PCR. (B) The expression of Ngal mRNA was detected by RT-PCR. Results are expressed as the means ± SD (n=6), # P<0.001, vs. the control group; **P<0.01, vs. the cisplatin group and the indicated test group. Supplemental Figure 4. Schematic diagrams illustrating the mechanism of G-CSF/AMD3100 mobilizing bone marrow–derived stem cells rescues mice from Cisplatin-induced acute renal failure. Exogenous AMD3100 specifically blocks CXCR4 mediated SDF-1/CXCR4 interactions in bone marrow microenvironment, resulting in BMSCs snap out the bone marrow niche, mobilize to the peripheral blood, and homing into injured kidney. The BMSCs promote renal repair via improving renal tubular cells proliferation and regeneration, regulating apoptosis and inflammatory cytokines. [file 13287_2021_2268_MOESM1_ESM.docx]

**The Combination of G-CSF and AMD3100 Mobilizes Bone Marrow-derived Stem Cells to Protect Against Cisplatin-induced Acute Kidney Injury in Mice**

Zhi Chen^1^, Xiang Ren^1^, Ruimin Ren^1^, Yonghong Wang^2^, and Jiwen Shang^1*^^[[1]](#footnote-1)^

1. ^1^Department of Urology, Shanxi Bethune Hospital, Taiyuan 030032, China. ^2^Department of Neurosurgery, Shanxi Bethune Hospital, Taiyuan 030032, China.

**Supplementary figure 1**


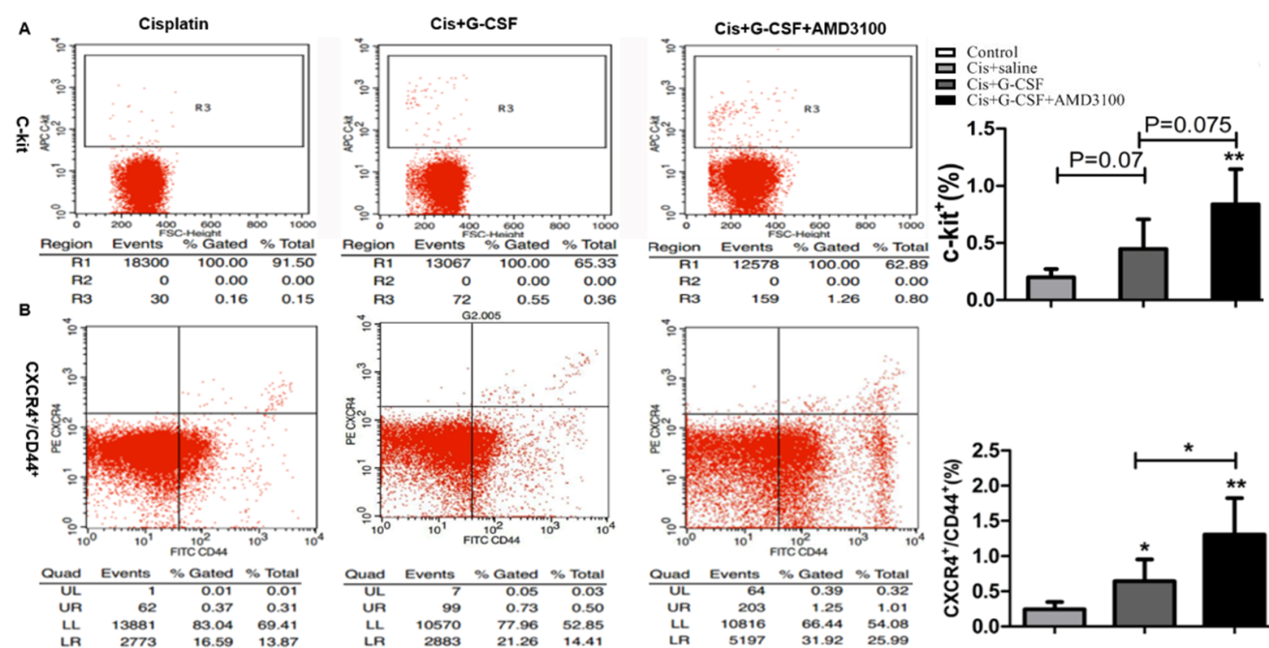


Supplemental Figure 1. Effects of G-CSF and/or AMD3100 on mobilization of stem cells. The percentage of c-kit^+^ cells (A) and CXCR4^+^/CD44^+^ cells in the circulating blood was performed flow cytometry analysis after induction of AKI in control cisplatin, G-CSF and G-CSF/AMD treated mice. Results are expressed as the means ± SD (*n=*5 per group),*P<0.05, **P<0.01, vs. the cisplatin group, and the indicated test group.

**Supplementary figure 2**

**
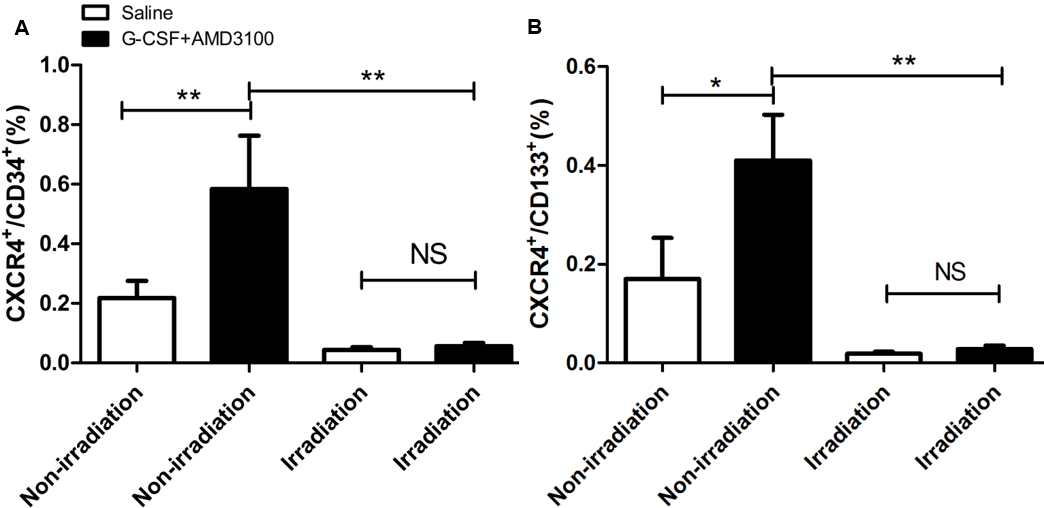
**

Supplemental Figure 2 Effect of bone marrow ablation (BMA) and G-CSF/AMD3100 treatment in C57BL/6J mice on mobilization of stem cells in the peripheral blood. Irradiated C57BL/6J mice received treatment with G-CSF/AMD3100 or saline, as described in the Materials and Methods section. 96 hours after the last injection of cytokines, collected of peripheral blood. The percentage of CXCR4^+^CD34^+^ cells (A), CXCR4^+^CD133^+^ cells (B) in the circulating blood was performed flow cytometry analysis. Results are expressed as the means ± SD (*n=*5 per group),*P<0.05, **P<0.01, NS : no significance.

**Supplementary figure 3**


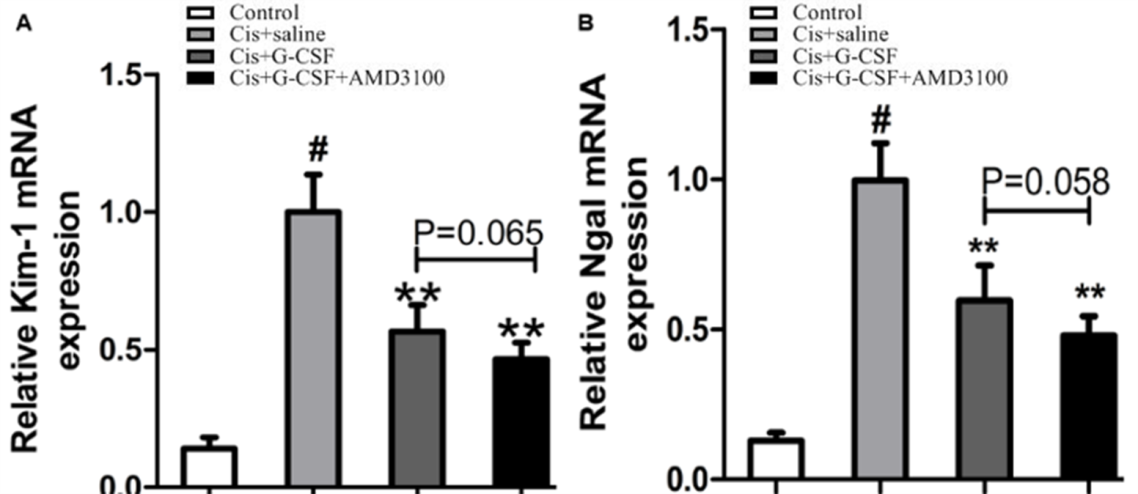


Supplemental figure 3 Effects of G-CSF/AMD3100 on the Kim-1, Ngal mRNA expression. (A)The expression of Kim-1 mRNA was detected by RT-PCR. (B) The expression of Ngal mRNA was detected by RT-PCR. Results are expressed as the means ± SD (*n=*6), # P<0.001, vs. the control group; **P<0.01, vs. the cisplatin group and the indicated test group.

**Supplementary figure 4**


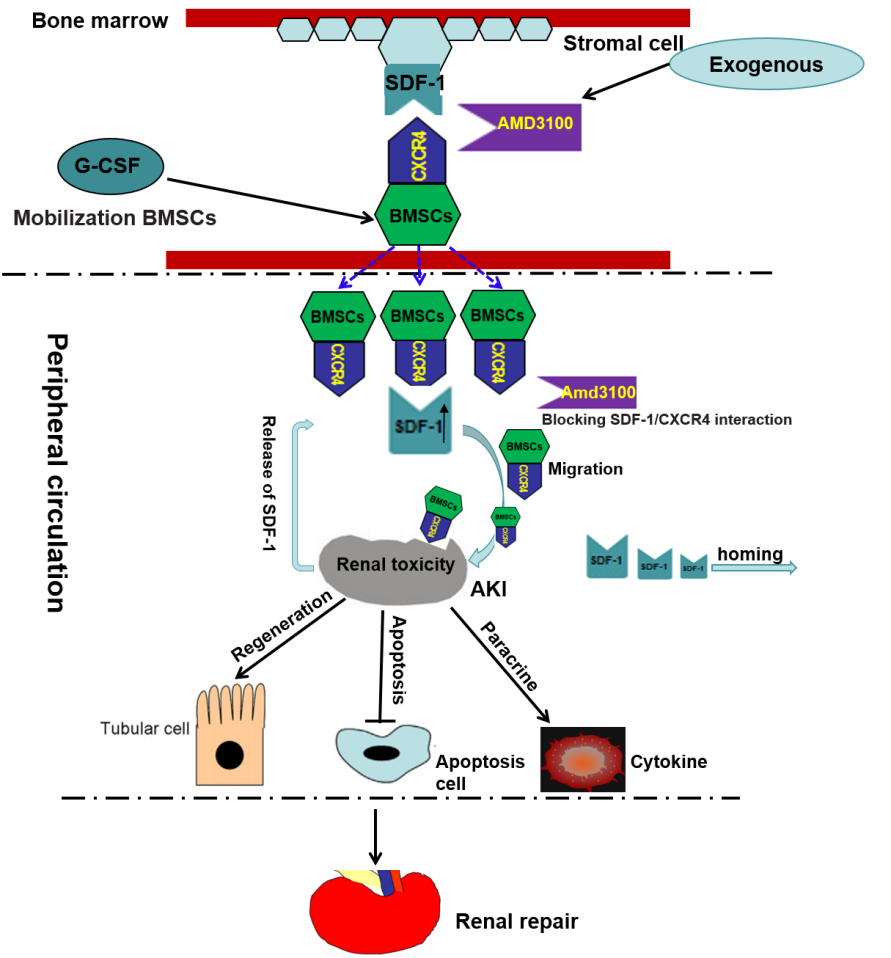


Supplemental figure 4 Schematic diagrams illustrating the mechanism of G-CSF/AMD3100 mobilizing bone marrow–derived stem cells rescues mice from Cisplatin-induced acute renal failure. Exogenous AMD3100 specifically blocks CXCR4 mediated SDF-1/CXCR4 interactions in bone marrow microenvironment, resulting in BMSCs snap out the bone marrow niche, mobilize to the peripheral blood, and homing into injured kidney. The BMSCs promote renal repair via improving renal tubular cells proliferation and regeneration, regulating apoptosis and inflammatory cytokines.

1. *Correspondence: sjw139@126.com

   Department of Urology, Shanxi Bethune Hospital, No. 99 Longcheng Street, Taiyuan 030032, Shanxi, China

   Full list of author information is available at the end of the article [↑](#footnote-ref-1)
